# Multimodal defect analysis and application of virtual machining for solid-state manufactured aluminium structure

**DOI:** 10.1007/s40964-024-00904-6

**Published:** 2024-12-10

**Authors:** Vladislav Yakubov, Halsey Ostergaard, Shishira Bhagavath, Chu Lun Alex Leung, James Hughes, Evren Yasa, Mani Khezri, Sandra K. Löschke, Qing Li, Anna M. Paradowska

**Affiliations:** 1 School of Civil Engineering, The University of Sydney, Sydney, NSW Australia; 2 Australian Nuclear Science and Technology Organisation, Kirrawee, NSW Australia; 3 Department of Mechanical Engineering, University College London, London, UK; 4 Advanced Manufacturing Research Centre (AMRC), University of Sheffield, Sheffield, UK; 5 Research Complex at Harwell, Harwell Campus, Oxfordshire, UK; 6 Sydney School of Architecture, Design and Planning, The University of Sydney, Sydney, NSW Australia; 7 School of Aerospace, Mechanical and Mechatronic Engineering, The University of Sydney, Sydney, NSW Australia

**Keywords:** Additive friction stir deposition, Solid-state additive manufacturing, Defects, Microstructure, Hardness

## Abstract

Additive friction stir deposition (AFSD) is an emerging solid-state non-fusion additive manufacturing (AM) technology, which produces parts with wrought-like material properties, high deposition rates, and low residual stresses. However, impact of process interruption on defect formation and mechanical properties has not yet been well addressed in the literature. In this study, Al6061 aluminium structure with two final heights and deposition interruption is successfully manufactured via AFSD and characterised. Defect analysis conducted via optical microscopy, electron microscopy, and X-ray computed tomography reveals > 99% relative density with minimal defects in centre of the parts. However, tunnel defects at interface between substrate and deposit as well as kissing bonds are present. Edge of deposit contains tunnel defects due to preference for greater material deposition on advancing side of rotating tool. Virtual machining highlights the ability to remove defects via post-processing, avoiding mechanical performance impact of stress concentrating pores. Electron backscatter diffraction revealed regions with localised shear bands that contain 1–5 µm equivalent circular diameter grains. Kissing bonds are exhibited in areas separated by large grain size difference. Meanwhile, Vickers hardness testing reveals hardness variation with deposit height. This work advances the understanding of complex microstructure development, material flow, and mechanical behaviour of AFSD Al6061 alloy.

**Supplementary Information:**

The online version contains supplementary material available at 10.1007/s40964-024-00904-6.

## Introduction

Additive friction stir deposition (AFSD) is a solid-state additive manufacturing (AM) process in which material is added layer-by-layer to form a part, based on its CAD model. Unlike fusion-based AM processes such as powder bed fusion (PBF) [1] or directed energy deposition (DED) [2], AFSD [3–5] is a deformation/pressure-based process. It utilises a rotating tool that directs feedstock material through its centre, using frictional heat and high pressure to extrude and bond the material layer by layer. The material remains below its melting point throughout the process, eliminating fusion-based defects such as lack of fusion porosity and solidification cracking. AFSD offers important environmental advantages such as direct recycling capabilities [6–9], and it is suitable for fabricating large structures since it is reported to have the highest deposition rate of all AM processes [4]. Despite its advantages, AFSD is still under development because of questions regarding flash formation, defect distribution, and variation in mechanical properties along the deposit height due to the complex AFSD thermal history [4, 10–12].

During AFSD, a deposit may be temporarily interrupted for many reasons such as inserting a new feedstock bar, staff breaks during manufacturing a large structure, or machine malfunction during print. When the process is interrupted, the deposited layers onto the substrate are cooled down, unless thermal control is implemented [13]. The impact of such temporary deposit interruption has not yet been investigated; defects may form at the interface of the first layer deposited after resumption if the AFSD parameters are not changed [8] Inadequate thermal input leads to poor bonding between the deposited material and substrate due to insufficient material mixing and flow during AFSD [14].

Internal defects and surface roughness can deteriorate mechanical properties, including fatigue life, leading to premature failure [15–20]. For example, irregular-shaped pores with sharper curvature have a greater impact than spherical pores [21–23]. While defects in AFSD of aluminium alloys have not been fully characterised yet, defects in friction stir welding (FSW), which is a solid-state welding process that shares similarities with AFSD [4, 24], have been comprehensively studied. In FSW, kissing bonds are defined as areas with separation of the materials or absence of metallic bonding and are caused by insufficient material stirring and low heat input [25, 26]. Meanwhile, flash is characterised as excess material extrusion as a ribbon-type structure and is induced in FSW by low tool traverse speed and high tool rotation speed, leading to material overheating, softening, and expulsion [27, 28]. Tunnel defects, also known as wormhole defects, are an internal cavity formed along the tool traverse direction caused by improper plasticisation of the material and deficient material movement around the tool pin [29, 30]. Flash and kissing bonds have been previously noted for AFSD [14, 31], although X-ray tomography techniques have not yet been applied to characterise shape, size and distribution in 3D of tunnel defects in AFSD [29]. The combination of optical microscopy and X-ray computed microtomography (XCT) can provide detailed visualisation of both internal defects and surface topography [32].

Age hardenable aluminium alloys (2000, 6000, and 7000 series) undergo precipitation strengthening by impeding the dislocation movement [33, 34]. During AFSD of Al6061 alloy, temperature during deposition was sufficient to dissolve alloying elements [35]. During structure build-up, elevated temperature may activate diffusion processes; however, local temperature and holding time are indirectly influenced, e.g. the first layers may be held at an elevated temperature for relatively longer than the final layers due to prolonged heat input from the AM process. Achieving homogeneous peak ageing requires strict control over temperature and holding time, which cannot currently be achieved during AM, thus leading to undesirable precipitation and hardness gradients in the AM structure [35, 36]. Although hardness cannot be directly correlated with mechanical properties such as tensile strength and ductility, areas with high hardness generally exhibit higher strength than areas with low hardness [37, 38]. Such hardness gradients lead to inconsistent mechanical properties across the manufactured structure.

In this research, scanning electron microscopy (SEM), energy-dispersive X-ray spectroscopy (EDS), and electron backscatter diffraction (EBSD) are applied to determine the microstructure of AFSD-manufactured Al6061 parts. Optical microscopy and XCT are used to reveal defect location and geometry in AFSD-manufactured Al6061 structure at uninterrupted and interrupted regions: (i) start (plunge point), (ii) steady state (in-plane movement area), and (iii) turnaround (out-of-plane layer transition). Vickers hardness testing is used to determine microhardness distribution at uninterrupted start areas as well as interrupted steady-state regions. A virtual machining analysis was performed in the steady-state sections of the Al6061 part using XCT to achieve structure that is almost porosity-free. The results will provide a guide for the machining of AFSD parts for pore minimisation.

## Materials and methods

### AFSD manufacturing

A commercially available MELD L3 machine located at the Advanced Manufacturing Research Centre North West (AMRC NW) of the University of Sheffield (UK) was used to deposit Al6061-T6 9.5 × 9.5 mm^2^ rod feedstock onto a 25 mm-thick machined Al6061-T6 substrate. Datasheet material composition for Al6061-T6 feedstock and substrate is provided in Table 1.

**Table 1:** Datasheet material composition for Al6061-T6 material used as AFSD feedstock and substrate

Si	Mg	Fe	Cu	Mn	Ti	Zn	Cr	Al
0.4–0.8	0.8–1.2	≤ 0.7	0.15–0.4	≤ 0.15	≤ 0.15	≤ 0.25	0.04–0.35	Balance

**Fig. 1:**
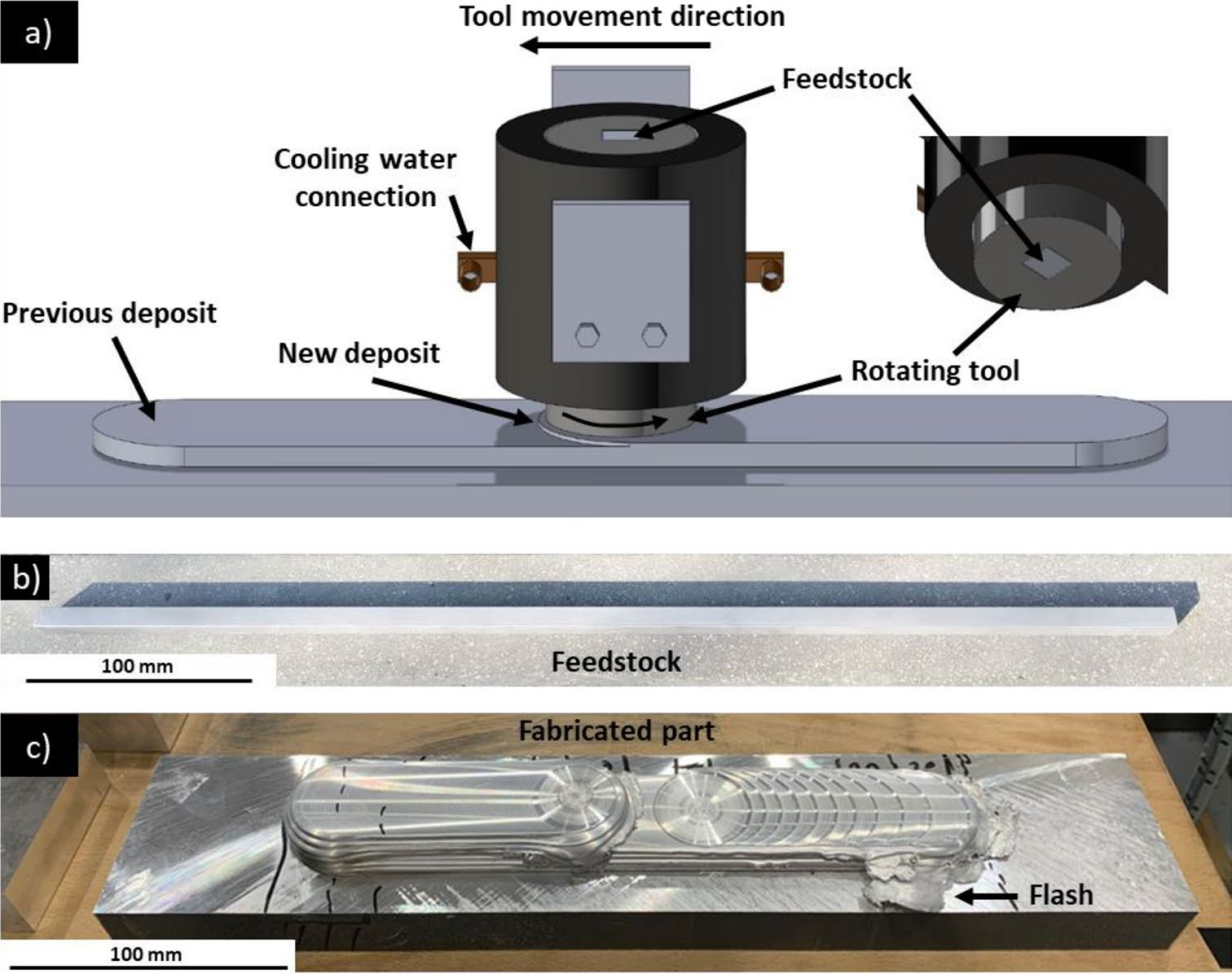
**a** Computer rendering of MELD L3 AFSD rotating tool and deposit in-process; **b** consumable feedstock used for sample fabrication; **c** complete Al6061 structure manufactured via AFSD

A 38 mm diameter AFSD rotating tool with a flat contact face was employed, and 3D render of the MELD L3 machine is provided in Fig. 1a. Feedstock rod is shown in Fig. 1b, and as-manufactured structure is shown in Fig. 1c.

Deposition parameters are described in Table 2. A bi-directional travel path was employed, e.g. once the rotational tool reached the end of the deposit, it would stop, raise by the target layer height, and then reverse its traverse direction.

**Table 2:** Summary of AFSD parameters used for Al6061 structure manufacturing

Identifier	First layer height (mm)	Subsequent layer height (mm)	Deposit height (mm)	Deposit length (mm)	Layer count	Feedstock feed rate (mm/min)	Tool in-plane movement speed (mm/min)	Tool out-of-plane movement speed (mm/min)
First deposit	0.5	1	10.5	222	11	152	381	8.9
Second deposit	1	1	8	89	8			

The initial deposit height was 11 layers. After a 20-h pause to ensure structure returned to ambient temperature, the deposition restarted on one half to deposit an additional 8 layers (19-layer total height). This allowed for the comparison of defect formation, microstructure, and mechanical properties between the interrupted and non-interrupted sections. No thermal management, e.g. preheating, active cooling, or PID control was employed during the process.

### Sample preparation

**Fig. 2:**
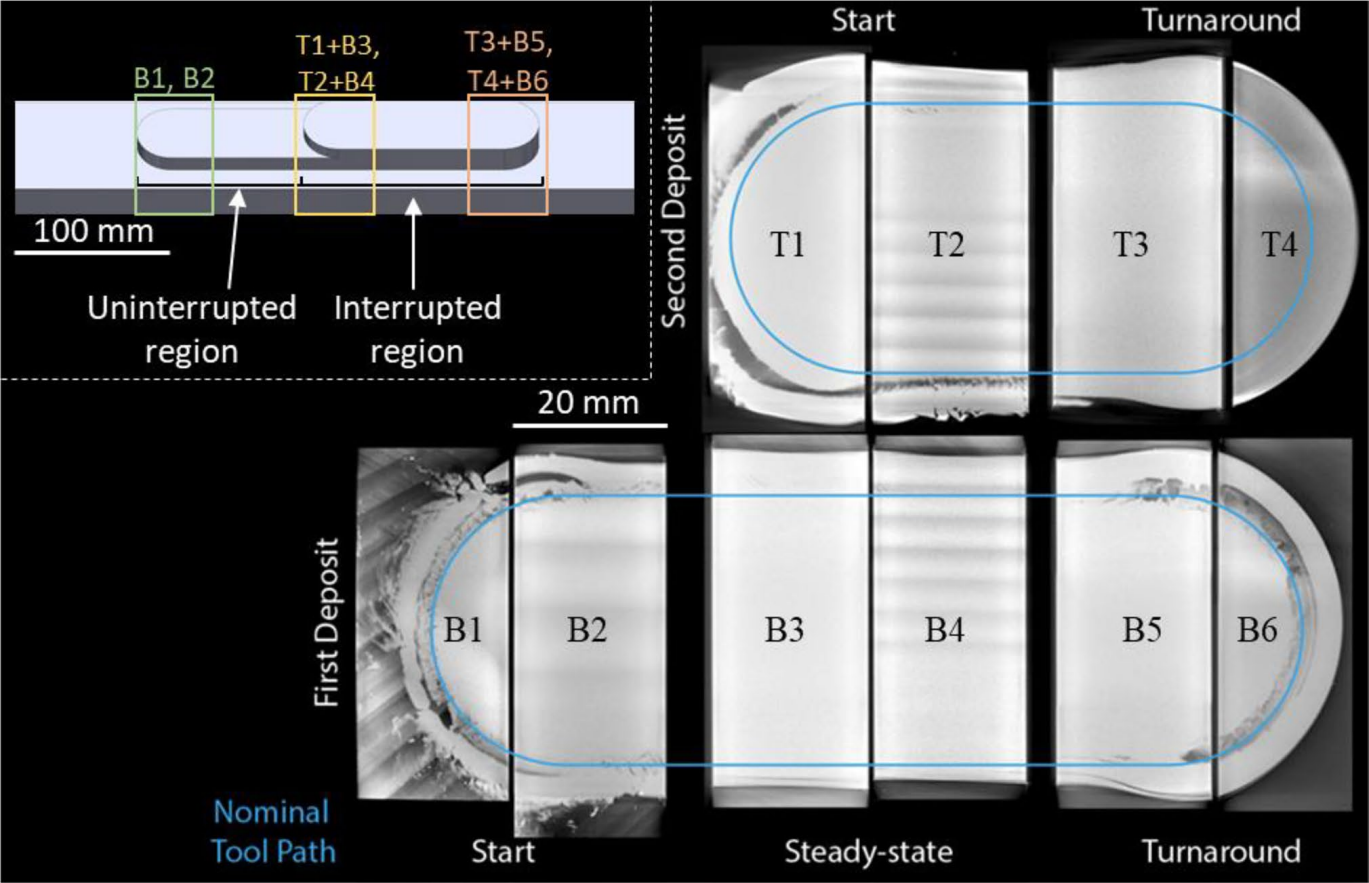
Individual section naming of investigated AFSD-manufactured Al6061 structure. Sections B1, B2, B3, B4, B5, and B6 indicate the first deposit, which includes uninterrupted sections B1 and B2. T1, T2, T3, and T4 indicate second deposit, which are made on top of B3, B4, B5, and B6, respectively, and consist of single parts where the deposition interruption is virtually segmented in XCT to identify defects caused by deposition interruption

The as-manufactured samples were labelled and cut as shown in Fig. 2 using a Secotom-10 precision cutting machine. Prior to hardness testing, the sample surface was wet ground with silicon carbide paper down to 4000 grit, and final polishing was conducted using 0.05 µm oxide polish suspension. For SEM–EDS and EBSD analysis, the polished samples were further ion-polished for 1 h at 6 keV and 2 h at 2 keV via Gatan PIPS-II.

### Microstructure analysis

Optical microscopy was conducted via Hirox MXB-050Z to examine surface defects. High-resolution SEM EBSD scans were conducted via Zeiss UltraPlus analytical FESEM with Aztec EBSD system (20 kV tension, high current mode, 120 µm aperture, 10–15 mm working distance, 0.45–1.7 µm step size), while elemental characterisation was performed using SEM–EDS in Zeiss Evo 50 with Aztec EDS system (20 kV tension, 30 µm aperture, 13–15 mm working distance).

### Hardness testing

To prevent local strain hardening effect on the hardness map, hardness indentation was performed on the polished sample surface with 1 mm spacing between each indent (equivalent to 4 × the indent size) using Struers DuraScan-80 automated Vickers hardness tester with 500 g force for 10 s (HV_0.5_). Each point was manually inspected, and anomalous readings were discarded.

### X-ray computed microtomography (XCT)

To reveal the defects in 3D, selected samples were scanned by Phoenix VTomex 160 CT machine equipped with a 160 kV X-ray source. The XCT scans were performed using scan parameters presented in **Supplementary Table 1**. Image slices were reconstructed using Datos|x software (Phoenix|X-ray), resulting in an image matrix of 1000 × 1000 × 1000 pixels^3^. To achieve high effective pixel resolution, each section was divided into 2 regions of interest and then scanned separately. After that, image registration and stitching was performed to combine both scans prior to further data analysis.

The stitched images were first filtered by a 2-pixel radius median filter and then segmented using random forest baser classifier in Labkit [39] a Fiji ImageJ [40] plugin. Avizo 2023 was used for volume rendering of the segmented images. The porosity quantification steps were carried out following the protocol depicted in [41] and pore metrics were measured using Morpholibj [42] plugin in Fiji ImageJ.

## Results

### Optical defect analysis

**Fig. 3:**
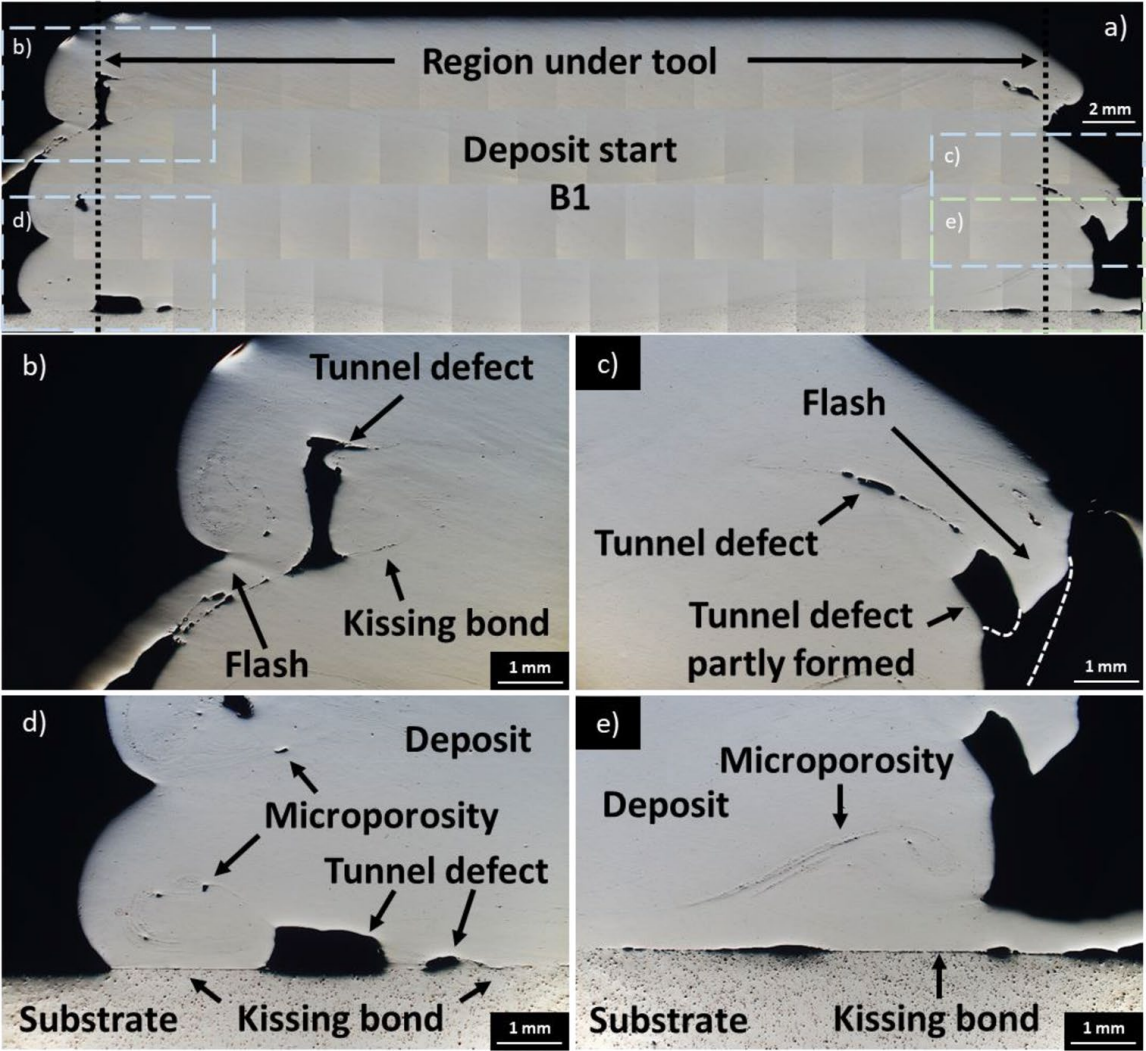
**a** Overview of AFSD deposit start point (section B1) with smaller boxed areas representing zoomed images (**b–e**); **b** Edge section near the top left of the sample demonstrating tunnel defect, flash presence, and region with kissing bond; **c** right edge section in the middle of the sample demonstrating tunnel defect, flash, and material overhang (tunnel defect precursor); **d** substrate interface on the left edge with tunnel defect, the deposit has kissing bond with the substrate; **e** substrate interface on the right edge with microporosity and kissing bond with the substrate

Figure 3a presents an overview of the initial deposit in the start area (section B1). This uninterrupted deposit contains well-bonded layers with no cracks, voids, or lack of bonding occurring in the region approximately 5 mm inward from the tool. The chosen processing parameters were suitable for both the initial bonding of the feedstock material to the substrate and for subsequent multilayer build-up. Some defects are present in the unconstrained region that is not under the rotating tool.

**Fig. 4:**
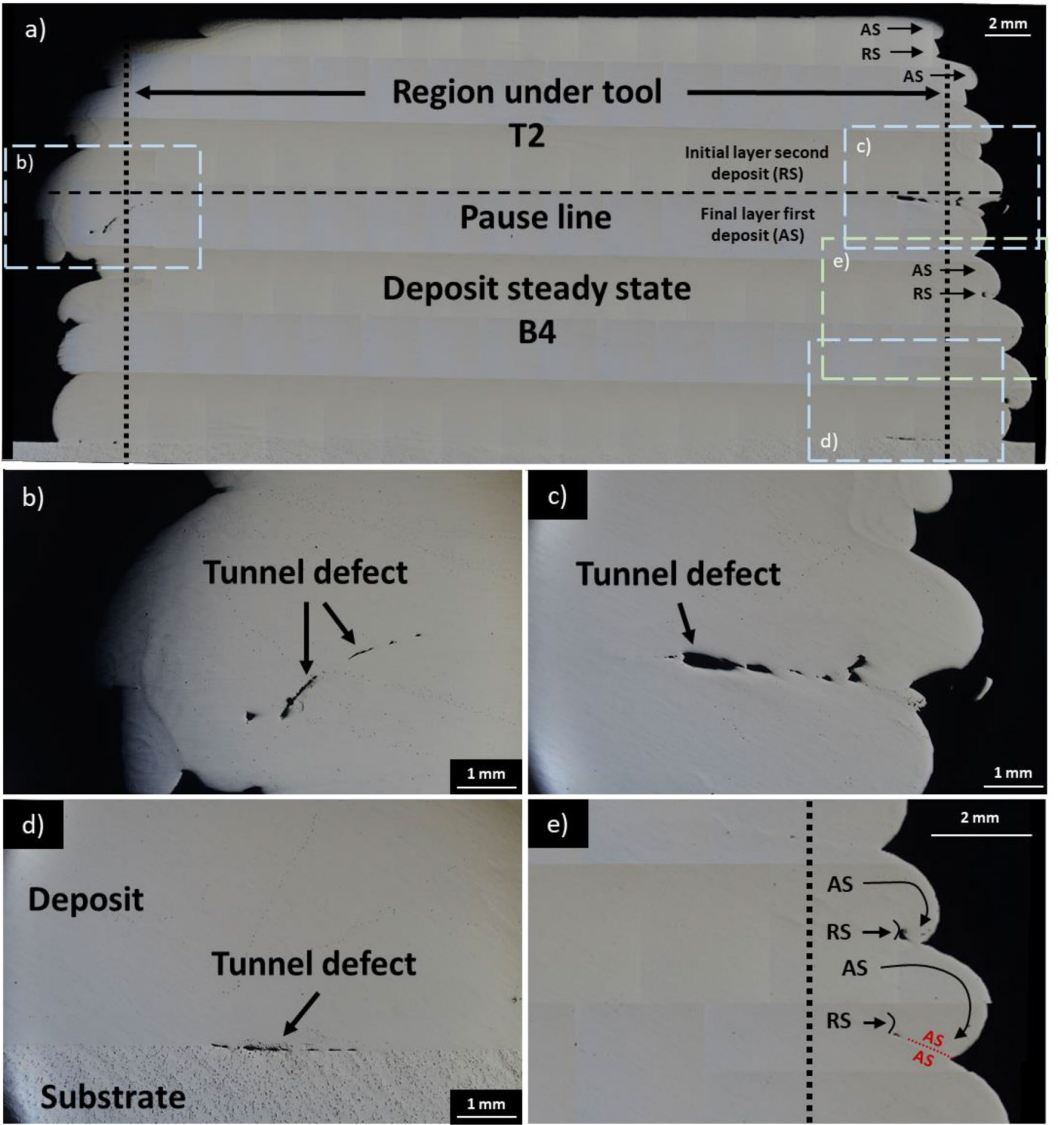
**a** Overview of AFSD deposit steady-state region (section T2 + B4) with smaller boxed areas representing zoomed images (**b–e**); **b** Left edge section at deposit interruption point demonstrating tunnel defect; **c** right edge section at deposit interruption point demonstrating tunnel defect; **d** right area of deposit, substrate interface with tunnel defect; **e** greater material extrusion on AS of deposit compared to RS of deposit, with red line demonstrating contact of two AS extrusions

Figure 3b demonstrates flash, tunnel defect, and kissing bonds. Tunnel defects other than those at the interface between deposit and substrate become apparent when the material is extruded beyond the preceding layers during multilayer build. The flash and tunnel defects in Fig. 3b, c are caused by material flow differences on the advancing side (AS) and retreating side (RS) of the rotating tool (Fig. 4e, discussed in Sect. 4.1).

As expected at the start point, large cavities (confirmed to be tunnel defects via XCT, see Sect. 3.2), porosity, and kissing bonds are observed between substrate and deposit in Fig. 3d, e at the tool edge. Near the tool centre, such defects are not present, and the deposit is well bonded to the substrate.

Figure 4a shows an overview of the interrupted deposit in the first deposit continuous movement region (section B4) and second deposit start point (section T2). The AS of the rotating tool achieves greater material push-out compared to RS.

As depicted in Fig. 4b, c, the tunnel defects are observed at the start point of the second deposit. Compared to the first deposit start point (section B1, Fig. 3d, e) and second deposit start point (section T2, Fig. 4b, c), defects at the interface between the substrate and deposit for section B4 (Fig. 4d) are considerably smaller and extend up to 2 mm inwards of the rotating tool edge. Similar to the observation made for section B1 (Fig. 3a), no defects are visible near the rotating tool centre and the area appears well bonded to the substrate.

In Fig. 4e, AS of deposit is seen to extrude further beyond the tool edge compared to RS of deposit. Because of this, material extruded on AS curls over the void created by shorter RS extrusion, thus promoting defect formation.

**Fig. 5:**
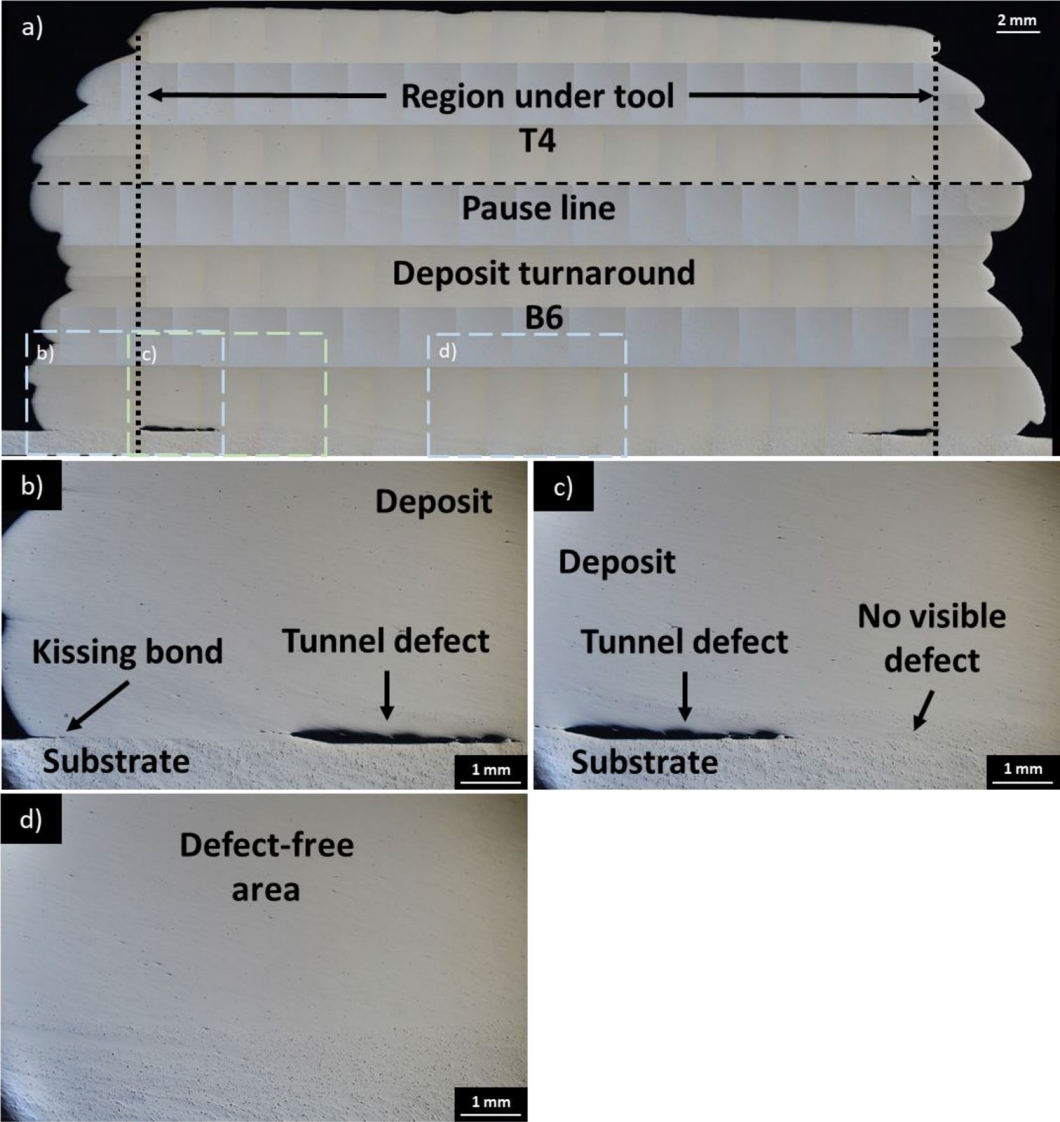
**a** Overview of AFSD deposit turnaround region (section T4 + B6) with smaller boxed areas representing zoomed images **(b–d)**; **b**) left edge, deposit and substrate interface with tunnel defect and kissing bond; **c**) left area of deposit, deposit and substrate interface with tunnel defect; **d**) deposit and substrate interface

Figure 5a presents the interrupted deposit at turnaround point (sections T4 and B6). In comparison to Fig. 4a, the interface between the second (section T4) and first deposits (section B6) is free of tunnel defects and appears to be well bonded even in the area near the tool edge.

In contrast, the interface between the first deposit and substrate (Fig. 5b**)** reveals kissing bond at the immediate edge of the deposit and a tunnel defect that extends approximately 3 mm inward from the tool edge. However, no defects are visible beyond the termination point of the tunnel defect as shown in Fig. 5c, d, and excellent deposit and substrate bonding is noted in Fig. 5d.

### XCT defect analysis

**Fig. 6:**
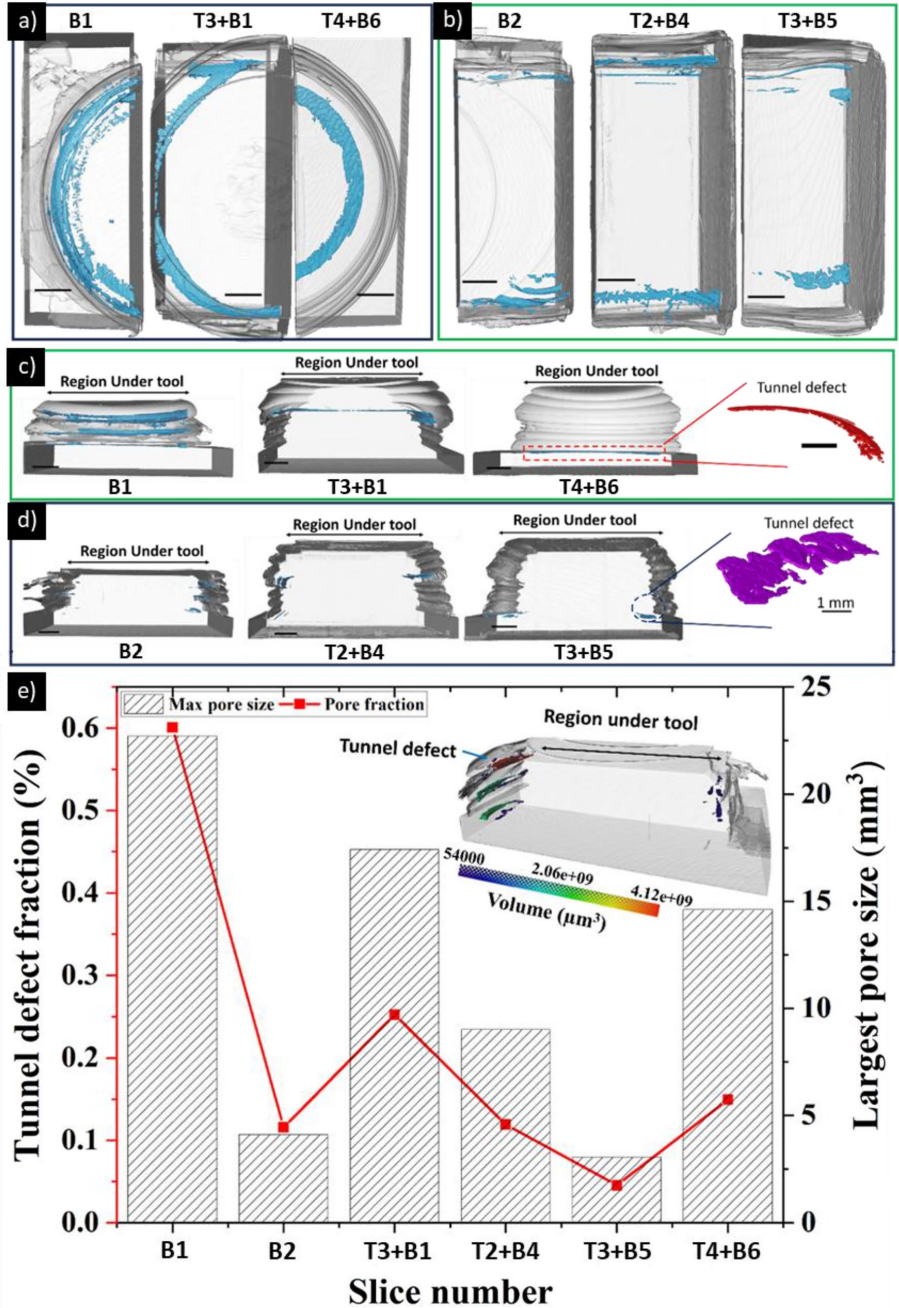
Top view 3D rendering of sample sections at **a** start and turnaround points and **b** steady-state region; front view of 3D rendering of sections at **c** start and turnaround points and **d** steady-state region; **e** 3D porosity fraction and largest pore size measured by XCT for six sections showing that the major contributor for total porosity fraction in the largest pore. Pores less than 5 voxels in equivalent circular diameter were not considered. Unlabeled scale bars provided in **a–d** correspond to 5 mm

Further to the optical microscopy analysis, the fraction and size of the largest pores are examined and quantified in 3D using XCT. Figure 6a–e shows volumetric rendering of investigated sections of the AFSD Al6061 structure wherein the blue features indicate the tunnel defects. Sections B1, T1 + B3, and T4 + B6 were grouped together (Fig. 6a) as they show typical porosity at the start, finish, and turnaround regions whereas sections B2, T2 + B4, and T3 + B5 represent the steady-state porosity (Fig. 6b). Figure 6c shows the presence of tunnel defect (zoomed structure in red inset) at the interface between deposit and substrate. Figure 6d displays a typical morphology of the tunnel defect that is located near the edge of the deposit (zoomed structure in purple inset). Figure 6e indicates the start and turnaround sections (Fig. 6a) exhibit a pore fraction > 0.15% and have the largest pore size of > 15 mm^3^. In contrast, the stead-steady sections have a pore fraction < 0.1% and have the largest pore size of < 10 mm^3^.

**Fig. 7:**
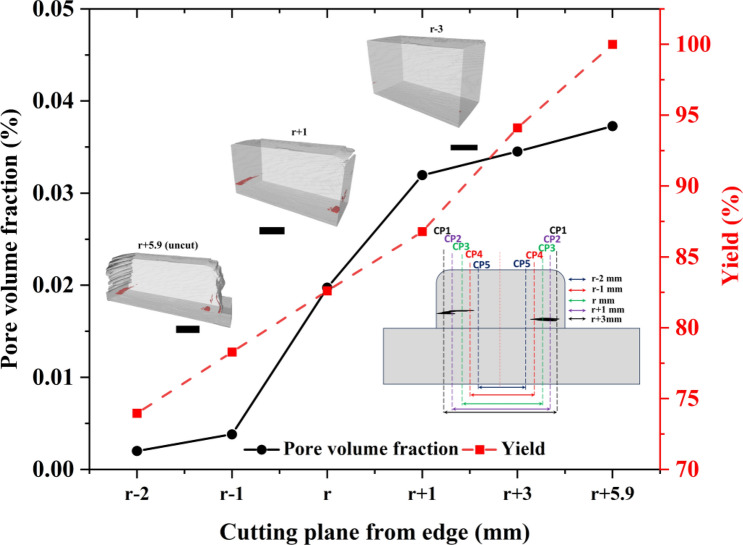
Virtual machining analysis for section T3 + B5. Pore volume fraction and yield are provided for machining *r-2* to *r* + *5.9* mm, where *r* represents rotating tool edge. Insets show the cutting plane for virtual machining and volume renders of virtually machined samples. Cutting plane is symmetrical with respect to rotating tool centre. All scale bars correspond to 9 mm

Porosity fraction and size/morphology of largest pore are detrimental to mechanical properties, especially fatigue strength. As the larger tunnel defects are located along the edges, they can be machined, thus potentially improving the mechanical and fatigue strength. In Fig. 7, virtual machining analysis of steady-state section T3 + B5 is presented. The machining is done in 5 virtual configurations and the machined areas are shown in Fig. 7 inset. To achieve pore-free sample, machining is conducted to a plane that is *r-2* mm, where *r* is the rotating tool edge, thus achieving a material yield of approximately 75%. However, it should be noted that the amount of machining can be adjusted according to defect requirements to achieve higher yield. Similar machining analysis for curved section T4 + B6 was performed as shown in supplementary Figure S1.

### Kissing bond analysis and microstructure

**Fig. 8:**
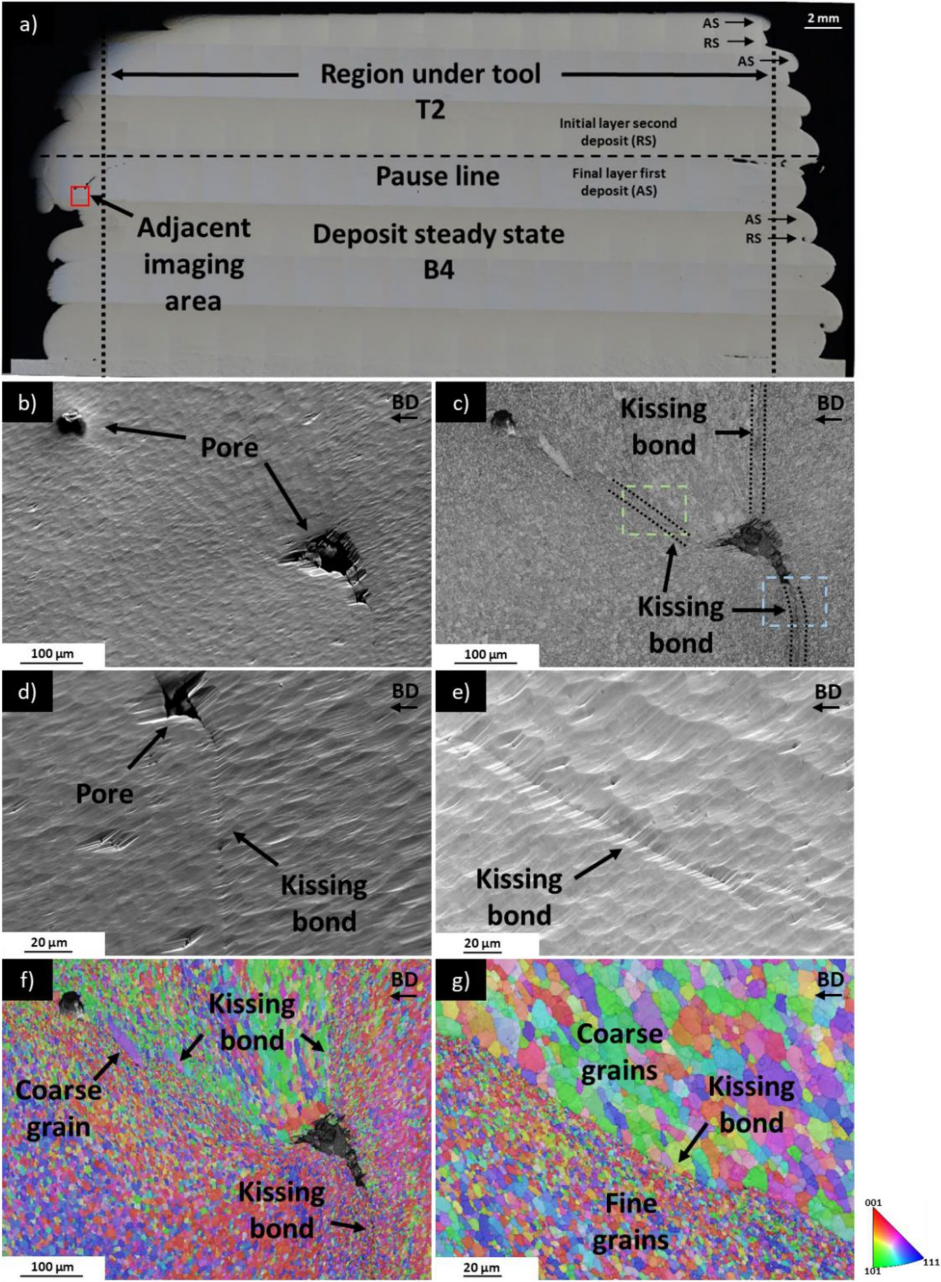
**a** Overview of AFSD deposit steady-state region (section T2 + B4), which is the adjacent cutting plane from which samples were extracted for **b–g**; **b** SEM image showing two pores in AFSD deposit (ion polishing causes sloping at pore edges); **c** band contrast (BC) image of **b**, with blue box indicating imaging area for **d** and green box indicating imaging area for **e**; **d** Kissing bond, with defect size on submicron scale; **e** Kissing bond, with defect size on submicron scale (ion polishing causes sloping at kissing bond); **f** EBSD of b) demonstrating grain size difference between the bonding layers; **g** EBSD of **e** demonstrating grain size difference between the two bonding layers

Not all the AFSD manufacturing defects are discernible through optical microscopy or XCT. Figure 8b (an SEM image) reveals two pores located near the deposit edge of steady-state section (located on adjacent cutting plane of section T2 + B4, which is shown in Fig. 8a). Although SEM image in Fig. 8b initially appears free of defects other than porosity, the band contrast (BC) in Fig. 8c exposes dark lines parting the two sides of the kissing bond. Figure 8d and 8e offers a magnified SEM image of the ion-polished surface at interfaces clearly indicating the presence of kissing bonds. Kissing bonds remained elusive to detection via optical microscopy or XCT, but Fig. 8f, g demonstrates the utility of EBSD in discerning the difference in grain size between the separated sections of the kissing bond [20, 21, 26].

**Fig. 9:**
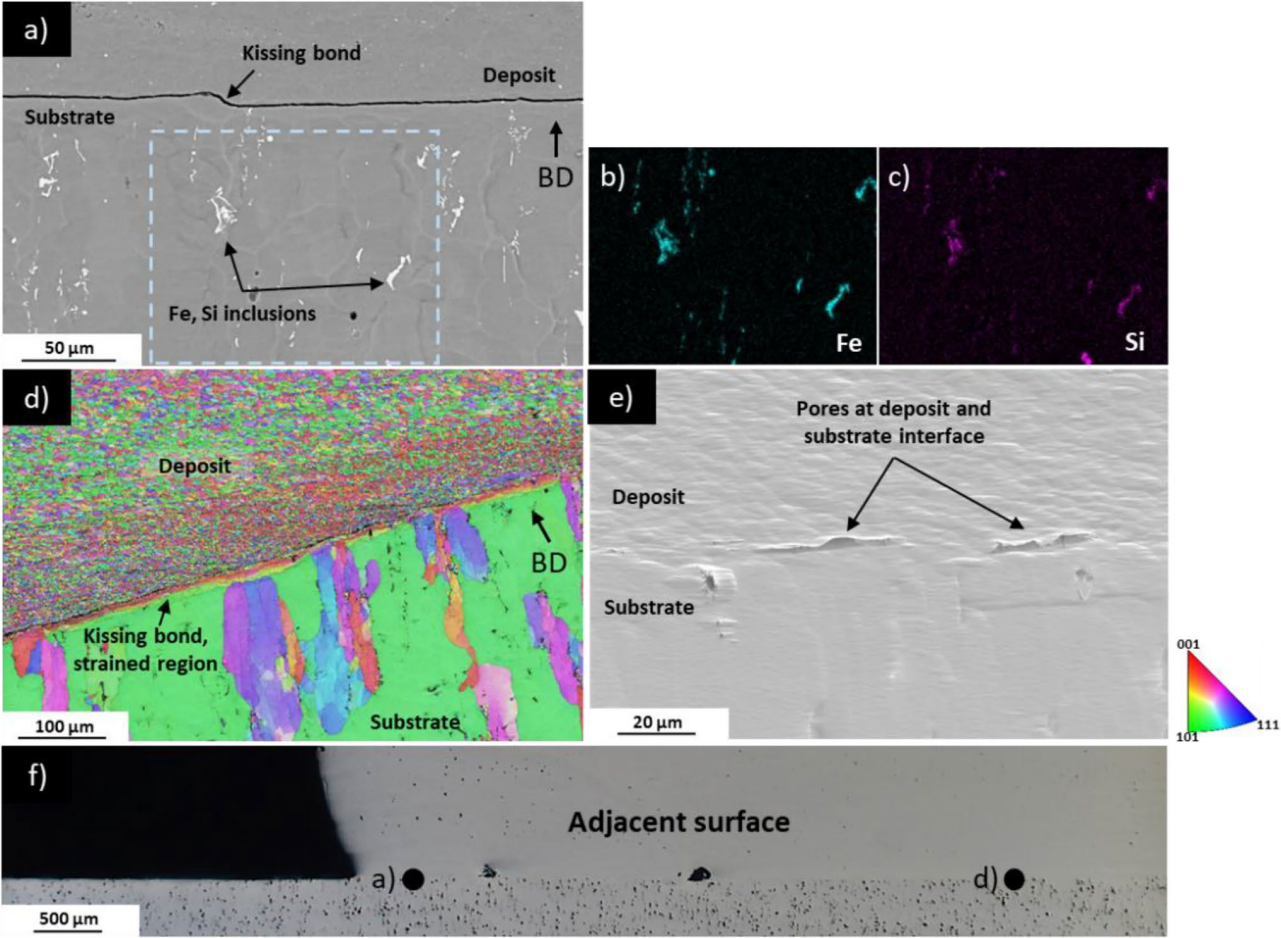
**a** SEM image of kissing bond at deposit and substrate interface near edge of deposit, white objects in substrate are Fe, Si inclusions; **b** EDS of boxed area in **a** showing presence of Fe; **c** EDS of boxed area in **a** showing presence of Si; **d** EBSD of **a** showing refined grains in deposit and coarse grains in substrate with strained region at interface; **e**) SEM image of pores at deposit and substrate interface approximately 5 mm from edge of deposit; **f** representative sample showing adjacent imaging area of **a** and **d**

In the optical image of the interrupted deposit within the steady-state region (sections T2 + B4), no defects other than tunnels are visible. However, the SEM image of sample extracted from adjacent cutting surface of section T2 + B4 (Fig. 9a, location in Fig. 9f) reveals a separation between the deposit and the substrate at the immediate deposit edge. Inclusions have been identified via EDS mapping as shown by the presence of Fe (see Fig. 9b) and Si (see Fig. 9c). These phases are likely to be Al(MnCrFe)Si, which formed due to the minor Mn, Cr, and/or Fe content in Al6061 [35, 43]. Large agglomerations of this phase seen in substrate are not observed in the AFSD deposit possibly due to the extreme deformation that causes breakup of oxides and intermetallics [44].

The EBSD analysis in Fig. 9d reveals that the grain size of the AFSD deposit is finer (1–5 µm in equivalent circular diameter [ECD]) than that of the substrate (several hundred micrometres ECD) and the interface clearly delineates these two areas.

The substrate side of the interface exhibits a significant amount of strain, which is assumed to be a result of the compressive force applied during AFSD. Approximately, 5 mm inward from the deposit edge in Fig. 9e, the deposit appears to be well bonded to the substrate. However, small pores with dimensions of approximately 20 µm in length and 2 µm in width are evident at the interface.

**Fig. 10:**
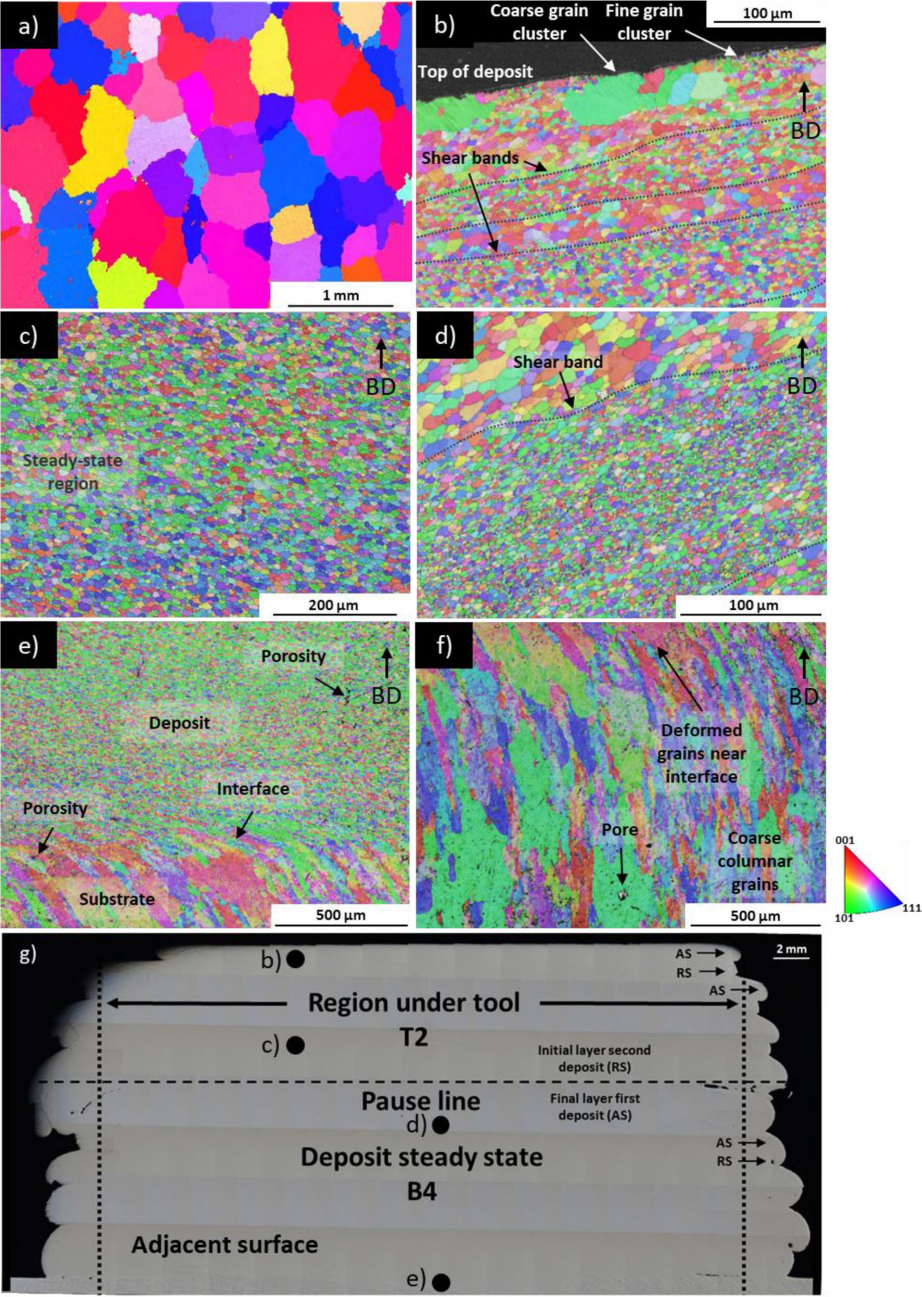
EBSD images of **a** feedstock rod; **b** top of deposit; **c** top-middle of deposit; **d** centre of deposit at shear line formed between first deposit and second deposit after interruption; **e** centre of deposit at substrate interface; **f** substrate. Image showing EBSD locations on adjacent cutting plane is provided as **g**

Even though plastic deformation during AFSD leads to refined grains in deposit (see Fig. 10b-10e, location in Fig. 10g) relative to feedstock (see Fig. 10a, grains several hundred micrometres ECD), the overall grain size distribution is heterogenous in the steady-state AFSD deposit. At final layer of the second deposit (Fig. 10b), the top surface contains clusters of coarse grains (20–70 µm ECD), which are neighboured by clusters of fine grains (1–5 µm ECD). Approximately, 80 µm from the deposit surface, grain structure consists of 5–10 µm ECD grains, while beyond this, shear bands containing 1–5 µm ECD grains are noted.

Further into the second deposit (Fig. 10c), a steady-state region exhibits 5–15 µm ECD grains and no shear bands. At the interface between the first and second deposit in Fig. 10d, a shear band approximately 150 µm wide contains 1–5 µm ECD grains.

The interface between the substrate and deposit as seen in Fig. 10e, f reveals a significant difference in the grain size and geometry between the two regions. The substrate grains shown in Fig. 10f are columnar and are oriented towards the build direction, which is incidental and a result of the substrate manufacturing process. During AFSD, the compressive force applied to feedstock bar causes penetration into the substrate and mixing of the two materials. In Fig. 10f, an area spanning approximately 500 µm is characterised by strained substrate grains orientated in the direction of material flow as well as breakup of the substrate grains, forming 5–20 µm ECD grains with same geometry as other areas of the deposit.

### Hardness distribution

**Fig. 11:**
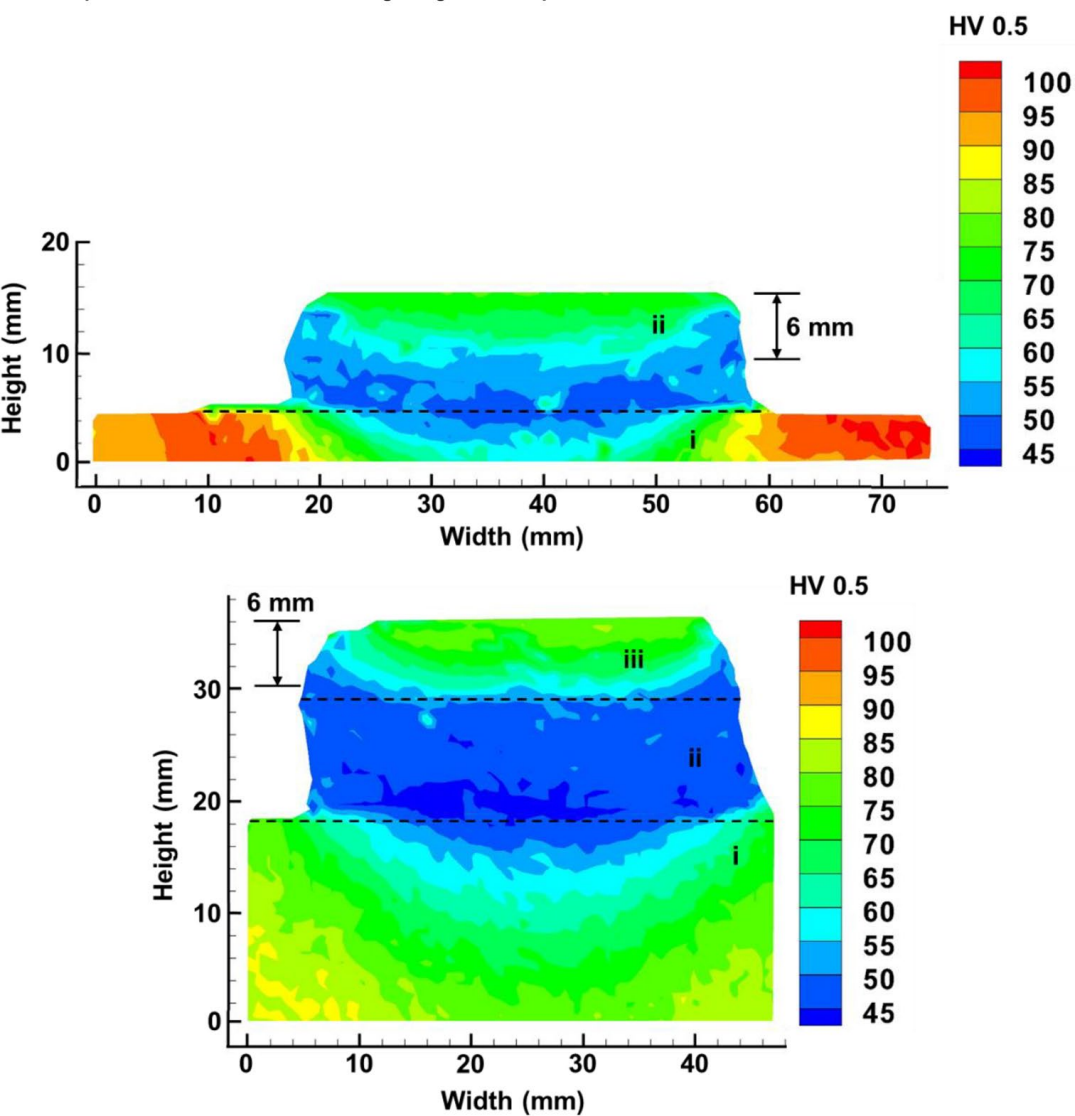
Hardness distribution of **a** section B1 and **b** section in between T2 + B4 and T3 + B5. Area (i) indicates substrate, (ii) indicates first deposit, (iii) indicates second deposit

Vickers hardness for the first deposit (Fig. 11a) and interrupted deposit containing the first and second deposits (Fig. 11b) reveal a trend where hardness is highest in substrate and lowest in the middle of deposit just above the substrate fusion line, reaching a maximum value of 105 HV_0.5_ and a minimum value of 47 HV_0.5_, respectively in Fig. 11a and a maximum value of 95 HV_0.5_ and a minimum value of 42 HV_0.5_, respectively in Fig. 11b. In both cases, the region within 6 mm of the top of deposit at centreline demonstrates intermediate hardness of approximately 75 HV_0.5_, while a low hardness region is present 5 mm into the substrate from the fusion line at centreline. The low hardness region overlaps with the substrate penetration and mixing region as presented in Sect. 3.1.

## Discussion

### Defect formation

Defects present near and beyond the tool edge are a result of feedstock interaction with the substrate and rotating tool. During AFSD, tool rotation along with force applied on the feedstock results in mixing of the deposit and substrate, forming a metallurgical bond [14, 45, 46]. However, the applied force under the rotating tool is non-uniform. The material under the rotating tool centre is in a compression-dominated state, since the feedstock is being forced downwards while the rotating tool remains at a set height [32, 47, 48]. This favours mixing with the substrate and causes the substrate to bulge and surge into the rotating tool face [48, 49], generating tunnel defects at the substrate and deposit interface in all sections.

As the material is extruded and moves away from the extrusion hole, it then becomes shear-dominated [32, 47]. The non-uniform force under the rotating tool is noticeable where the region near the deposit centreline, corresponding with the centre of the tool, experiences good mixing with the substrate, resulting in excellent bonding (Fig. 5a). Meanwhile, the areas towards the tool edge are not well bonded (Fig. 5b, c). A rotating tool with protrusions will likely result in different behaviour in this regard [50].

At the start of the deposition process, feedstock and substrate are initially at ambient temperature and the underside of the rotating tool is not saturated with deposit material. To achieve sufficient material extrusion to fill the void between the rotating tool and substrate, heating of the feedstock to cause softening must occur first [51]. A compressive force applied to feedstock material during tool rotation results in volumetric heat generation via deformation and interfacial heat generation via friction at the substrate interface [32], and the rotating tool typically does not begin the traverse until it is sufficiently saturated. Dwell time at this stage provides the opportunity for overfeeding [51], resulting in a large amount of flash at the start area (Figs. 1c and 3b, c) relative to other sections of the deposit.

Figure 4e shows that the deposit on the AS of the tool is pushed out further than the deposit on the RS. This asymmetry has been previously reported for AFSD-manufactured Al-alloy structures [48, 51] and was attributed to higher material temperature on AS due to frictional heating, shear force inducing material flow in direction of tool rotation, and influx of material from RS to AS. Meanwhile, Jin et al. [52] simulated the interaction of Al6061 with the AFSD tool and found material under rotating tool AS was under tensile stress, while under rotating tool RS, it was under compressive stress. Figures 3b, c and 4b, c show tunnel defects elongated tangent (Figs. 3c and 4c) and normal (Figs. 3b and 4b) to the substrate top surface. These defects are caused by layer overlap during asymmetric AS and RS material extrusion.

Although XCT analysis shows that all AFSD sections achieve > 99% relative density, these measurements do not include pores smaller than the resolvable resolution of the XCT scan (see Sect. 2.5). Nonetheless, the concern is identifying relatively large pores > 150 µm, which provides useful data to end-users allowing for the development of machining solutions to remove these critical pores in the ASFD parts (see Fig. 7).

Given that the large tunnel defects are located at the edge of rotating tool, the edges can be machined to minimise the detrimental impact of these pores on the mechanical performance. Here, virtual machining of steady-state sections was performed in 5 different configurations (see details inset of Fig. 7). The analysis shows the possibility to achieve pore-free sample by machining to a plane that is *r-2* mm as discussed in Sect. 3.2. An overall production yield of approximately 75% is achieved, which can be further optimised in future studies.

### Grain structure and material flow

The AFSD process involves thermo-mechanical phenomena, resulting in plastic deformation during material deposition, and the temperature of an aluminium feedstock under the rotating tool has been experimentally measured to be in the range of 76%-92% of the feedstock melting point [32]. As such, the deposited material not only undergoes substantial plastic deformation but is also exposed to elevated temperatures (cyclically), establishing the fundamental requirements for dynamic recovery (DRV), geometric dynamic recrystallisation (GDRX), and static grain growth [53].

In FSW [54, 55], friction stir processing (FSP) [54, 56], and AFSD [31, 49], plastic deformation and heat generation will induce dislocations. For materials with a high stacking fault energy, like aluminium [57], elevated dislocation density triggers their rearrangement via slip and climb mechanisms [53, 54, 58, 59]. This, in turn, diminishes the material’s strain energy and initiates DRV, forming equiaxed subgrains with nearly dislocation-free interiors. Furthermore, prior boundaries respond to subgrain boundary interface tensions and become serrated, and when boundary separation approaches subgrain size, GDRX occurs [31, 53, 54]. Therefore, deposit in as-manufactured condition consists of recrystallised strain-free grains; however, these grains will grow if the material remains above its annealing temperature [31, 53, 55].

Due to insufficient compressive force for layer mixing, kissing bonds near the tool edge and outside of the tool edge are observed to have significantly different grain sizes on either side of the defect (see Figs. 8f and 9d). The kissing bonds contain finer grains relative to other locations of the deposit. For example, the deposit grains at the substrate interface in Fig. 9d are 1–5 µm ECD. Meanwhile, deposit grains at the stirred area under the tool shown in Fig. 10e are coarser at 5–20 µm ECD. This can be explained by the DRV, GDRX, and static grain growth mechanisms. Since the linear velocity of the tool head increases as the distance from the rotation centre increases, areas at the edge of the deposit experienced relatively higher intensity shearing [32, 47], favouring plastic deformation-dependent grain refinement mechanisms. Simultaneously, the temperature in these areas is lower compared to the region near the rotating tool centre [47, 51], suppressing static grain growth.

At the top of the deposit in Fig. 10b, 20–70 µm ECD grains are neighboured by clusters of 1–5 µm ECD grains, and a similar situation is seen in Fig. 8f at a kissing bond at the edge of the deposit. Tang et al. [31] also observed this phenomenon but only at a kissing bond on the 9th layer of an 18-layer AFSD-manufactured Al6061 structure. This was attributed to two factors: the first is energy stored in sub-grain boundaries driving sub-grain growth, and the second is consumption of neighbouring fine grains that completely recrystallised. Despite clusters of coarse grains neighbouring fine grains at the top layer and at a kissing bond at edge of deposit, shear bands containing 1–5 µm ECD grains are noted approximately 100 µm from the deposit surface in Fig. 10b and at layer interface in Fig. 10d. Such shear bands were found at layer interface by both Tang et al. [31] and Perry et al. [48]; however, these areas are characterised by relatively high temperature and persistent thermal cycling [32, 51]. Therefore, the role of high-temperature exposure and thermal cycling on grain growth requires further investigation.

### Impact of deposition interruption on hardness distribution

It is known that thermal cycling during AFSD manufacturing results in lower strength and hardness for precipitation hardened Al alloys, including Al6061 [12, 35, 60–62]. Figure 11a, b shows that regardless of deposition interruption, the hardness trends are similar. In either case, the region within 6 mm of the top of the deposit at the centreline demonstrates a hardness of approximately 75 HV_0.5_. Under this area, the hardness decreases until it reaches 45–55 HV_0.5_, coinciding with hardness expected for Al6061 in severely overaged or annealed conditions [63, 64]. Meanwhile, the hardness begins to increase approximately 5 mm into the substrate from the fusion line at the centreline. It can be concluded that hardness stabilises at 45–55 HV_0.5_ approximately 10 mm from the topmost layer, with this hardness region spanning previous layers and the area approximately 5 mm into substrate. Therefore, the deposition interruption is unlikely to affect the hardness distribution if the interruption occurred at least 10 mm from the topmost layer, since the hardness will have reached the stable value range of 45–55 HV_0.5_.

## Conclusion

A detailed material characterisation and analysis of an AFSD-manufactured Al6061 structure with deposition interruption were conducted to examine defect formation, grain structure, material flow, and hardness distribution. The results are summarised as follows:

1. Defects near rotating tool edge at substrate and deposit interface are attributed to low compressive force under rotating tool at tool edge and bulging of substrate material into rotating tool face, while greater material push-out on AS relative to RS results in tunnel defect formation when material is deposited onto previous layer.
2. Although the deposit achieves > 99% relative density, the presence of pores raises mechanical performance concerns due to these pores acting as stress concentrators. However, virtual machining along deposit width to distance equivalent to 2 mm inwards from tool edges eliminates these defects.
3. Grain size is influenced by thermo-mechanical action. While majority of deposit contains 5–20 µm ECD grains, shear bands which are ≤ 150 µm wide containing 1–5 µm ECD grains are noted. Furthermore, kissing bonds not detectable through optical microscopy or XCT may be present in regions which are separated by large difference in grain size.
4. Deposition interruption during AFSD has a negligible impact on hardness distribution in precipitation hardened Al6061 alloy since hardness stabilises at 45–55 HV_0.5_ approximately 10 mm from the topmost layer, regardless of interruption.
5. Process parameters and build geometry influence defect formation and hardness distribution. Research on process parameter management for minimisation of tunnel defects caused by deposition interruption as well as development of process parameters for retention or development of high hardness in precipitation strengthened Al alloys will be beneficial for the uptake of AFSD technology by industry.

## Supplementary Information

Below is the link to the electronic supplementary material.

Supplementary file1 (DOCX 509 KB)

## Data Availability

The raw data required to reproduce these findings are available upon request by contacting Prof Anna Paradowska by email anna.paradowska@sydney.edu.au.
