# Supplementary material for: Multimodal defect analysis and application of virtual machining for solid-state manufactured aluminium structure
Source: Prog Addit Manuf. 2024 Dec 10;10(8):5281–97. doi: 10.1007/s40964-024-00904-6 (PMC12267324; doi:10.1007/s40964-024-00904-6)
Supplement: Supplementary file 1 — Supplementary file1 (DOCX 509 KB) [file 40964_2024_904_MOESM1_ESM.docx]

**Supplementary information 1**

**Supplementary Table 1.** A summary of the XCT scanning parameters

| Part identifier | Voxel size (µm^3^) | Voltage (kV) | Current (µA) | Integration time (ms) | Number of projections | Number of images averaged/projection |
| --- | --- | --- | --- | --- | --- | --- |
| B1 | 24.986 | 100 | 90 | 1000 | 1000 | 3 |
| B2 | 30.06 | 100 | 90 | 1000 | 1000 | 3 |
| T1+B3 | 30.87 | 100 | 90 | 1000 | 1000 | 3 |
| T2+B4 | 30.06 | 100 | 90 | 1000 | 1000 | 3 |
| T3+B5 | 30.06 | 100 | 90 | 1000 | 1000 | 3 |
| T4+b6 | 30.06 | 100 | 90 | 1000 | 1000 | 3 |

**Supplementary information 2**


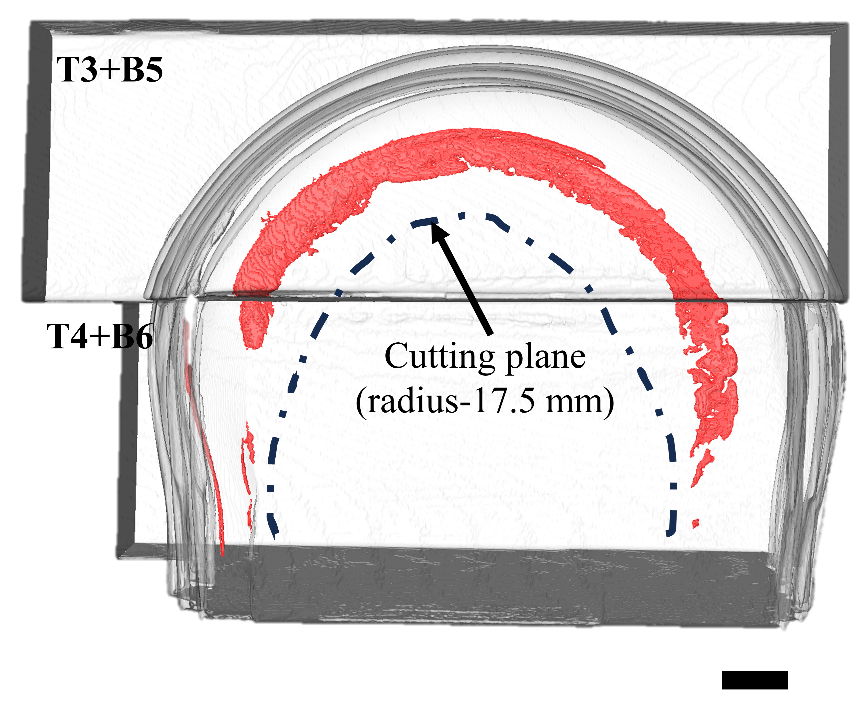


**Supplementary Figure S1.** Virtual machining analysis for section B5+T3 and B6+T4 (sections stitched) along the length. Scale bar corresponds to 5 mm.
